# Short-Term Bisphosphonate Therapy Could Ameliorate Osteonecrosis: A Complication in Childhood Hematologic Malignancies

**DOI:** 10.1155/2010/206132

**Published:** 2010-06-10

**Authors:** N. A. Greggio, M. Pillon, E. Varotto, A. Zanin, E. Talenti, A. C. Palozzo, E. Calore, C. Messina

**Affiliations:** ^1^Pediatric Endocrinology and Adolescence, Department of Pediatrics, Hospital-University of Padova, 35128 Padova, Italy; ^2^Hemo/Oncology, Department of Pediatrics, Hospital-University of Padova, 35128 Padova, Italy; ^3^Pediatric Radiology, Department of Pediatrics, Hospital-University of Padova, 35128 Padova, Italy; ^4^Pharmacy, Veneto Oncology Institute - IRCCS, 35128 Padova, Italy

## Abstract

Osteonecrosis (ON) is a critical complication in the treatment of childhood leukemia and lymphoma. It particularly affects survivors of acute lymphoblastic leukemia and non-Hodgkin lymphoma reflecting the cumulative exposure to glucocorticosteroid therapy. ON is often multiarticular and bilateral, specially affecting weight-bearing joints. A conventional approach suggests a surgical intervention even if pharmacological options have also recently been investigated. We reported two cases of long time steroid-treated patients who underwent Bone Marrow Transplantation (BMT) for hematological disease. Both patients developed femoral head osteonecrosis (ON) that was diagnosed by magnetic resonance imaging (MRI) and the ON was also accompanied with pain and a limp. Despite of the conventional strategies of therapy, we successfully started a short-term treatment with bisphosphonates in order to decrease the pain and the risk of fracture.

## 1. Introduction

Osteonecrosis (ON) is recognised as a complication of the treatment of hematologic malignancies in children and adolescents. The hips and the knees are the joints affected more frequently but involvement of the ankles is also described [1, 2]. The majority of patients present pain, limping, articular collapse, arthritis, and limitation of movement but in some cases it is completely asymptomatic. Spontaneous resolution can occur in these patients, especially in the case of small lesions [3] even if a majority of them have a progressive disorder, ending in the collapse of the affected joints. The Harris hip score [4], evaluating pain, functional capacity, range of motion and deformity, is very useful to asses morbidity.

Published reports and retrospective analysis are often limited to symptomatic patients. For these reasons, the true prevalence is probably underestimated [5].

In the last few years, the number of patient survivors after BMT has increased and the long follow-up period has made it possible to observe some late effects. ON is increasingly reported as a severe disabling complication, as well as osteoporosis, especially in patients receiving steroids for cancer treatment or as prophylaxis/treatment for graft-versus-hostdisease (GVHD) [6]. An early diagnosis of ON is essential in order to prevent its progression and MRI is considered the gold standard for early diagnosis and followup [7].

Several risk factors for ON have been identified in the LLA context. It seems that ON is more common in whites and in adolescents than in blacks [8–10] or children especially under 13 years old with a high BMI [9, 10]. The maturing bone of the adolescents may be more susceptible to the development of ON.

The pathogenesis is complex and includes suppression of bone formation, expansion of the intramedullary lipocyte compartment and a direct effect on the nutrient arteries. The Children Cancer Group (CCG) 1882 study also demonstrates that the incidence of ON correlates with the amount of dexamethasone received [10]. Corticosteroids are integral to the management of childhood acute lymphoblastic leukaemia (ALL) [11]. Improvements in event-free survival (EFS) have been achieved with the addition of dexamethasone to standard prednisone-based therapies [12]. This therapy has been associated with a dramatic increase in the occurrence of ON and may be directly linked with dexamethasone, which seems to be stronger than prednisone in both its antileukemic and toxic effects [11, 13].

We described the cases of two pediatric patients who were on long-term steroid treatment before BMT for hematologic disease and for acute or chronic GVHD. They presented ON of the femoral head and osteoporosis after BMT. Bisphosphonates (Alendronate) were used to treat them for about a period of 12 months, after parental consent, and the therapy ended when the pain and limping had stopped for a significant period of time.

## 2. Case  1

The first patient, now 20 years old, had originally been diagnosed with anaplastic Large Cell Lymphoma when he was 12 and received corticosteroid therapy according to the AIEOP NHL 92 protocol, Dexamethasone (DXM) 448mg/m^2^ until relapse and DXM 420 mg/m^2^ from the first relapse to the pretransplantation conditioning regimen. After 4 relapses, he underwent matched unrelated donor (MUD) transplantation. The conditioning regimen was TBI (1200 cGy), Thiotepa (TT), Ciclophosphamide (CPM), and Antithymocyte globulin (ATG).

**Figure 1:**
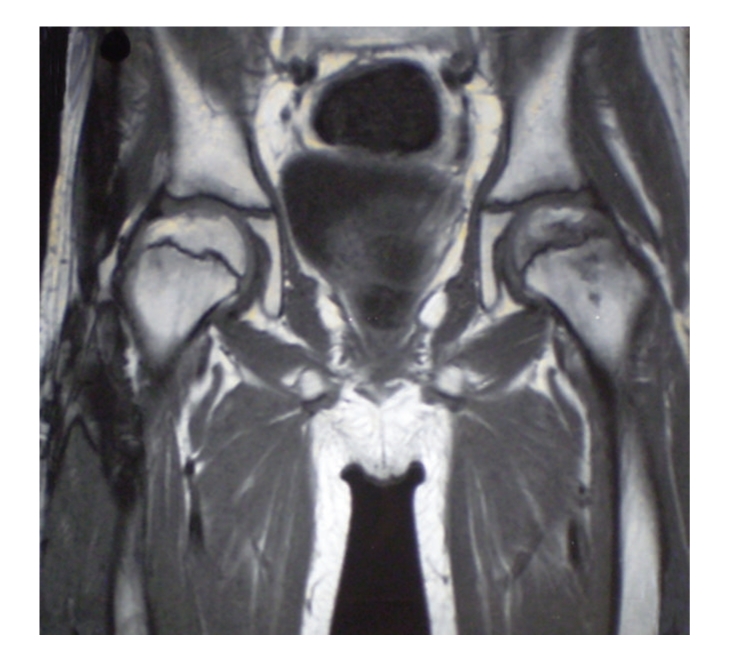
Moderate bilateral joint effusion and fat-like aspects of osteonecrosis involving the weight bearing portion of the femoral heads. In the left hip where the articular surface is partially flattened. Bone marrow oedema is also present bilaterally, around the femoral capital necrosis.

A six-month treatment with corticosteroids (Methylprednisolone 8975 mg/m^2^, Prednisone 10500 mg/m^2^, and Hydrocortisone 2710 mg/m^2^) was used for grade IV acute and severe chronic GVHD of gut, skin, and lungs. Eight months after BMT, while still under corticosteroid therapy, he developed a limp and severe pain in the left hip. An MRI was performed (Figure 1) and revealed a moderate joint effusion, as well as fat-like aspects of osteonecrosis involving the weight bearing portion of the femoral heads particularly extruded in the left hip where the articular surface was partially flattened. Bone marrow oedema was also present bilaterally around the right femoral capital necrosis (Figure 1). A DEXA was performed, which showed osteoporosis. The blood test indicated that related bony tissue turnover was normal.

**Figure 2:**
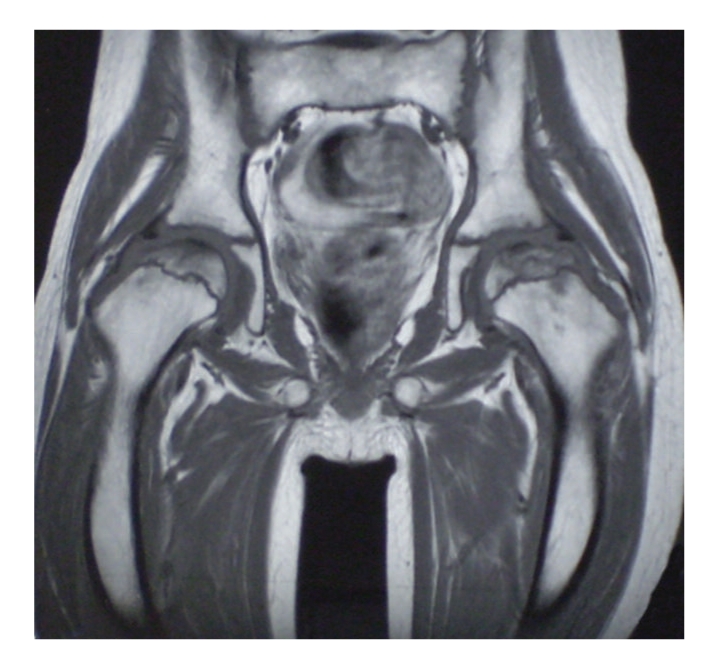
Bilateral joint effusion and bone marrow oedema are disappeared. The right femoral head shows a regular articular surface but with aspects of midollary sclerosis. Partial aspect of fragmentation on the left femoral capital necrosis.

Orthopaedic suggestions were to limit weight bearing, functional discharge, and the rest of hip joints. Since the pain persisted, a treatment with Alendronate was added to the conventional therapy when he was 13 years old. This therapy was started 6 months after ON had been diagnosed, at the dosage of 10 mg a day, and ended 11 months later, without any side effects. One month after the beginning of bisphosphonates therapy, the patient reported improvement of the limp together with a considerable decrease of pain and a gradual recovery of normal motor activity. An MRI, performed nine months from the diagnosis of ON, showed a resolution of the bilateral joint effusion and bone marrow oedema. The right femoral head showed a regular articular surface but with aspects of midollary sclerosis. The left femoral capital necrosis presented a partial aspect of fragmentation (Figure 2). 

At the last followup, 7 years from the initial symptoms, the patient reported the absence of pain as well as fully functioning joint motility.

## 3. Case  2

The second patient is an 18-year-old girl who had a bone marrow relapse for ALL at 10 and had undergone an MUD BMT. Pretransplantation conditioning regimen included TBI 1200 cGy, TT, CPM, and ATG. Before BMT, corticosteroids were administered according to induction and reinduction therapy of AIEOP protocol 9502 (Methylprednisolone 211 mg/m^2^, Prednisone 150 mg/m^2^, and DXM 360 mg/m^2^). In addition, Prednisone was administered for one month (total dose 1950 mg/m^2^) to prevent an acute GVHD. After 6 months from the bone marrow infusion, she developed a limp and pain on the weight bearing joint in the right hip.

**Figure 3:**
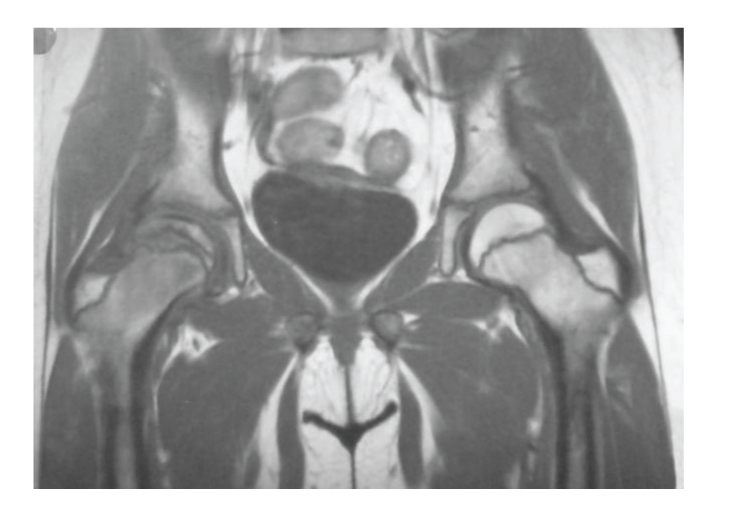
Flattened right femoral head, joint effusion, enlarged physes, bone marrow oedema of the femoral neck, and minimal sclerotic changes of the articular surface. There is a remarkable increase of the signal on T_1_W sequences only in the deep portion of the femoral head.

MRI examination showed a flattened right femoral head, joint effusion, enlarged physes, bone marrow oedema of the femoral neck, and minimal sclerotic changes of the articular surface. After Gadolinium IV, there was a remarkable increase of the signal on T_1_W sequences only in the deep portion of the femoral head (Figure 3).

A DEXA was also performed, which showed diminished bone-density, confirming the diagnosis of osteoporosis. The blood test indicated that related bony tissue turnover was normal.

**Figure 4:**
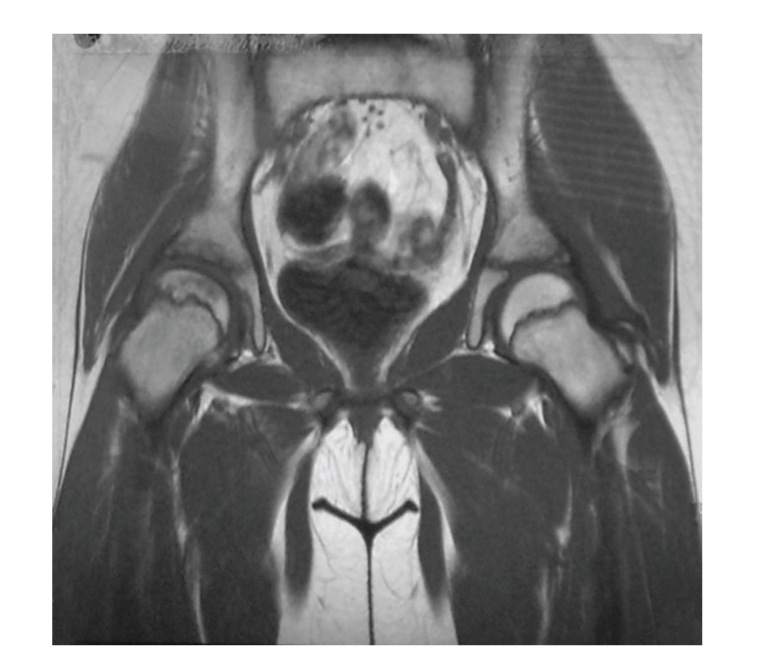
Boundary line of the necrosis of the capital of the femur with a fat-like aspect, good remodelling of the femoral head, and morphologic normalization of the physes are shown in this follow up MRI.

Pressure was relived from the affected joint, and the patient underwent physiotherapy. Six months after the diagnosis of ON, when she was 11 years old, she started a therapy with Alendronate, 5 mg a day for two weeks and then we increase the dose to 10 mg a day. Two months later, the patient reported a significant decrease of pain. Bisphosphonates therapy lasted for 12 months, without any side effect. The last MRI, performed 30 months from initial symptoms, showed a better boundary line of the necrosis of the capital of the femur with a fat-like aspect, good remodelling of the femoral head, morphologic normalization of the physes, and only minimal flattening of the articular surface (Figure 4). Bone-density densitometry results also improved. Now 6.5 years since the initial symptoms, she has completely recovered and is even capable of mild sport activities.

## 4. Discussion

Skeletal morbidity, characterized by bone pain, osteonecrosis, fractures, loss of mobility, bone deformation, or osteopenia, is frequently encountered in patients affected by ALL. Orthopaedic management of symptomatic ON varied. This morbidity can cause immobility and consequently a substantial reduction in the quality of life and may require surgical interventions such as core decompression [14], arthrodesis, and joint replacement. Furthermore, resurfacing hemiarthroplasties were recently performed to treat advanced osteonecrosis in young adult patients [15]. Alleviation of weight from the affected joints (employing crutches or wheelchair) for a certain period together with physical therapy is usually recommended for many patients. There are some concerns [16] regarding using surgical interventions in growing open physes, although the conventional approach is still surgery. Nonsurgical intervention includes external electrical stimulation/capacitance coupling [17, 18] and the use of hyperbaric oxygen [19].

We still have little experience concerning the pharmacological treatment of ON but recently some studies [20–23] have shown that Alendronate offers an added advantage in children and adolescents with either ALL or a malignant lymphoma with ON.

Bisphosphonates are very important inhibitors of osteoclastic bone resorption *in vivo* [24] and are used in diseases such as osteoporosis, hypercalcemia of malignancy, Paget's disease of bone, and osteolytic bone disease [25–27]. However, up to now there had not been any indications for the use of these drugs in the treatment of ON. They are safely used in treating childhood hypercalcemia, secondary to acute lymphocytic leukaemia [28, 29]. The administration of bisphosphonates produced normocalcemia in these patients apparently without any significant side effects. ON of the jaw or oesophageal cancer after bisphosphonates infusion represents well-known complications in adult cancer patients [30, 31] but in children these complications have not been reported. Long-term potential effects from their use during the active phase of growth still remain unknown.

Goldbloom and collaborators have also used bisphosphonates to treat vertebral fractures of two children affected by acute lymphoblastic leukemia (ALL) [32]. The authors report the successful use of pamidronate on both patients who had originally had pain and low bone mineral density. In addition to standard chemotherapy, pamidronate (1 mg/kg, IV) was given bimonthly. Initial rapid symptom relief and gradual improvement of bone mineral density were demonstrated in both patients. It remains to be demonstrated whether osteonecrotic collapse can also be prevented with the use of these medicaments. In fact, the inhibition of osteoclast activity by the use of bisphosphonates could possibly lead to an increase in bone mass and strength in these sites. Moreover, in orthopedic conditions it is often possible to apply the drug locally. This will give a much higher local oncentration because of the pharmacokinetics effects of the bisphosphonates which remain in the site for a long time. When we had originally decided to treat our patients with Alendronate, there was no information on this drug concerning its use in relation to ON. Therefore, in our patients the lack of symptoms and the absence of fractures after 7 years seem to be encouraging.

These promising approaches including the use of these antiresorptive drugs as well as strategies such as the employment of lipid lowering agents are being currently explored [33]. It is very difficult to prevent ON in children but high-risk groups of patients are identifiable and have to be monitored; clinical and radiological examinations have to be conducted as well as measures of functional assessment which would all help the patient attain a better quality of life.
